# ICOS Regulates the Generation and Function of Human CD4^+^ Treg in a CTLA-4 Dependent Manner 

**DOI:** 10.1371/journal.pone.0082203

**Published:** 2013-12-02

**Authors:** Jian Zheng, Ping-Lung Chan, Yinping Liu, Gang Qin, Zheng Xiang, Kwok-Tai Lam, David B. Lewis, Yu-Lung Lau, Wenwei Tu

**Affiliations:** 1 Department of Pediatrics and Adolescent Medicine, LKS Faculty of Medicine, the University of Hong Kong, Hong Kong SAR, PR China; 2 Division of Immunology and Allergy, Department of Pediatrics, School of Medicine, Stanford University, Stanford, California, United States of America; Agency for Science, Technology and Research (A*STAR), Singapore

## Abstract

Inducible co-stimulator (ICOS) is a member of CD28/Cytotoxic T-lymphocyte Antigen-4 (CTLA-4) family and broadly expressed in activated CD4^+^ T cells and induced regulatory CD4^+^ T cells (CD4^+^ iTreg). ICOS-related signal pathway could be activated by the interaction between ICOS and its ligand (ICOSL). In our previous work, we established a cost-effective system to generate a novel human allo-antigen specific CD4^hi^ Treg by co-culturing their naïve precursors with allogeneic CD40-activated B cells in vitro. Here we investigate the role of ICOS in the generation and function of CD4^hi^ Treg by interrupting ICOS-ICOSL interaction with ICOS-Ig. It is found that blockade of ICOS-ICOSL interaction impairs the induction and expansion of CD4^hi^ Treg induced by allogeneic CD40-activated B cells. More importantly, CD4^hi^ Treg induced with the addition of ICOS-Ig exhibits decreased suppressive capacity on alloantigen-specific responses. Dysfunction of CD4^hi^ Treg induced with ICOS-Ig is accompanied with its decreased exocytosis and surface CTLA-4 expression. Through inhibiting endocytosis with E64 and pepstatin A, surface CTLA-4 expression and suppressive functions of induced CD4^hi^ Treg could be partly reversed. Conclusively, our results demonstrate the beneficial role of ICOS-ICOSL signal pathway in the generation and function of CD4^hi^ Treg and uncover a novel relationship between ICOS and CTLA-4.

## Introduction

Inducible costimulator (ICOS), a member of CD28 and cytotoxic T lymphocyte antigen 4 (CTLA-4) cell surface receptor family, is absent in naïve human T cells but could be induced in activated human T cells [1]. Expressions of ICOS exert distinct impacts on the outcomes of CD4^+^ T cell-mediated immune responses corresponding to different immunological context. The effects of ICOS on the functions of T cell are mainly initiated by the interaction between ICOS and its ligand (B7 related protein 1, B7RP1 or ICOSL), which is generally expressed on diverse antigen presenting cells (APC) [2]. ICOS-related signal plays important roles in many T cell-mediated disease models, such as inflammation [3], anti-viral immune responses [4], anti-tumor immune responses [5], allogeneic grafts rejection [6,7], wound healing [8], and thus representing a potential target of treatment [9]. However, the roles of ICOS-ICOSL interaction in human immune system are still inclusive.

The roles of ICOS in T cell-mediated immune responses are multiplex. In addition to its roles in the proliferation and migration of T cells in mice [10,11], ICOS was also found to be involved in differentiation of murine CD4^+^ T cell recently. Under specific conditions, ICOS could either promotes Th2 cell differentiation [12] or mediate Th1-like responses [13]. The role of ICOS in Th17 cell differentiation remains controversial [14], although its expression in T cells was shown to attenuate Th17-related inflammation in an experimental autoimmune encephalitis (EAE) model [15]. More recently, ICOS was also identified as a critical marker of follicular T cells (Tfh), and its interaction with ICOSL on B cells was necessary for the development and antibody production of murine B cells [16]. Actually, the effect of ICOS on humoral immunity has been confirmed in common variable immunodeficiency patients [17].

CD4^+^ regulatory T cells (CD4^+^ Treg) represent for a small population in human peripheral lymphocytes and exhibit suppressive capability on distinct immune responses. CD25, Foxp3 and CTLA-4 are mostly accepted markers for CD4^+^ Treg, whereas the roles of ICOS in CD4^+^ Treg remain to be clarified. In murine system, ICOS was found to be an important marker of induced CD4^+^ Treg [18,19] and plays indispensible roles in induction and maintenance of immune tolerance besides its function in regulating the differentiation of effector T cells [20]. Moreover, murine CD4^+^ICOS^+^ Treg demonstrated better survival, proliferative, and even suppressive abilities than their ICOS^-^ analogues [21]. In support to this, ICOS knock-out NOD mice exhibits dominant defect in their CD4^+^ Treg [22]. Consistent with its beneficial role in the generation of murine CD4^+^ Treg [23-25], the expression of ICOS was recently found to promote the generation [26,27], drive the activation [28], and improve the function of human CD4^+^ Treg [26,29,30]. In clinic, ICOS^+^CD4^+^ Treg has been identified in type I autoimmune pancreatitis patients and found to help ameliorate the disease severity [31]. Interestingly, functions of both murine and human ICOS-related signals in T cells are found to be regulated by CD28 and CTLA-4 [4,13,29,32], whereas the effect of ICOS-ICOSL on the expression of CTLA-4 has never been illustrated. 

In our previous studies, we established a novel system to generate alloantigen-specific CD4^hi^ Treg from their naive precursors by co-culturing them with allogeneic CD40-activated B cells [33]. In this study, we examined the expression of ICOS in these induced CD4^hi^ Treg, and investigated the roles of ICOS-related signals in their generation and functions. It was found that ICOS-ICOSL promoted the generation of CD4^hi^ Treg through reducing the apoptosis of CD4^+^ T cells. More importantly, the blockade of ICOS-ICOSL by ICOS-Ig during induction impaired the suppressive capacity of generated CD4^hi^ Treg. The reduced suppressive capacity of CD4^hi^ Treg was due to down-regulated expression of its surface CTLA-4, which might resulted from decreased exocytosis caused by ICOS-Ig. By inhibiting the endocytosis of CTLA-4 with E64 and pepstatin A, the function of CD4^hi^ Treg could be partly reversed. Taken together, our results demonstrate the beneficial roles of ICOS in the generation of functional human CD4^+^ Treg and a novel relationship between ICOS and CTLA-4.

## Materials and Methods

### Ethics statement

Written consent for the use of buffy coat for research purposes was obtained from the donors by the Hong Kong Red Cross Blood Transfusion Services at the time of blood donation. The use of buffy coat for this experiment was approved by the Institutional Review Board of the University of Hong Kong/Hospital Authority Hong Kong West Cluster (IRB Reference Number: UW 07-390).

### Generation of CD40-activated B cells

B cells from PBMCs were stimulated via CD40 using NIH3T3 cells transfected with the human CD40 ligand (t-CD40-L cells) as described previously [33,34]. Briefly, PBMC were co-cultured with the lethally irradiated (96Gy) t-CD40-L cells in the presence of IL-4 (2.0 ng/ml; R&D systems, Minneapolis, MN, USA) and cyclosporine A (5.5×10^-7^ M, Sigma-Aldrich, St. Louis, MO, USA) in IMDM (GIBCO-BRL, Carlsbad, CA, USA) supplemented with 10% heat-inactivated human AB serum, 50 µg/ml transferrin (Boehringer Mannheim, Indianapolis, IN, USA), 5.0 µg/ml insulin (Sigma-Aldrich, St. Louis, MO, USA), and 15.0 µg/ml gentamicin (Life Technologies, USA) at 37°C in 5% CO_2_. After 14 days of co-culture, more than 95% of the viable suspended cells are routinely CD19 positive. For future use, B cells were cryopreserved in 10% DMSO FBS. For co-culture with CD4^+^ T cells, CD40-activated B cells were always Ficoll-density centrifuged followed by washing with PBS twice to remove nonviable cells including remaining t-CD40-L cells. 

### CD4^+^ T cell isolation

Human naïve CD4^+^ T cells were isolated from healthy donor PBMC by negative selection using a naïve CD4^+^ T cell isolation kit (depletes CD8, CD14, CD16, CD19, CD25, CD36, CD56, CD123, TCRγ/δ, CD235a, and CD45RO) (Miltenyi Biotec, CA, USA). The purity of selected cells was routinely more than 98% as determined by flow cytometric analysis. 

### Induction and expansion of CD4^hi^ Treg

Freshly purified human naïve CD4^+^CD45RO^-^CD25^-^ T cells were co-cultured with allogeneic CD40-activated B cells at a T cell: B cell ratio of 10:1 in RPMI 1640 medium supplemented with 10% heat-inactivated human AB serum. In repeated stimulation experiments, allogeneic CD40-activated B cells were added every 6 days of culture. Functional and phenotypical hallmarks of the induced and expanded T cells were examined at indicated time of culture. The expansion of viable cells was determined by counting aliquots of cells that excluded trypan blue dye.

### Flow cytometric assays

Cells were analyzed by flow cytometry using a FACSAria (BD Biosciences, CA, USA) after staining with the following fluorescence-conjugated mAb (all from BD-Biosciences unless otherwise specified): anti-human CD4-Alexa-405, anti-human CD25-APC, anti-human CD27-PE, anti-human CD44-PE, anti-human CD45RO-APC, anti-human CD63-FITC, anti-human CD107a-FITC, anti-human glucocorticoid induced TNF receptor-related protein (GITR)-PE, anti-human CTLA-4-PE, anti-human ICOSL-PE, and anti-human ICOS-PE. Intracellular staining was performed after cell fixation and permeabilization reagents (Fix and Perm) (BD Biosciences, CA, USA). The following anti-cytokine mAbs were used: anti-human IL-10-PE (R&D, CA, USA), anti-human TGF-β-PE (IQ Products, Netherlands) and their isotype-matched controls of irrelevant specificity. Human Foxp3 staining kit (eBiosciences, CA, USA) was used for Foxp3 staining. 

### Cell death and proliferation assays

Percentage of apoptotic and proliferative cells in the co-culture was determined by propidium iodide (PI) (Invitrogen Life Technologies, CA, USA) and carboxyfluorescein succinimidyl ester (CFSE) (Sigma) correspondingly as described previously [34]. For PI staining, cells were stained with PI for 15 minutes and analyzed by flow cytometry for PI-positive (dead) cells gating on the cell type of interest. For CFSE staining, 22 µl of 0.05mM CFSE solution were added into 1 ml of cells (concentration of 5×10^6^ cells/ml) and incubate at 37°C for 5 min. Stained cells were harvested at indicated time points and the levels of CFSE were analyzed using FACS Aria-II (BD) and Flowjo software (Tree Star Inc.).

### Mixed lymphocyte reaction (MLR) assays

The suppressive capacity of CD40-activated B cell-induced CD4^hi^ Treg was examined in a MLR system as described before [33,34]. CD4^hi^ Treg was sorted by a FACSAria after 9 days of co-culturing naive CD4^+^ CD25^-^ T cells and allogeneic CD40-acitvated B cells. The purity of sorted cells was routinely more than 95%. The sorted CD4^hi^ Treg referred as “regulator” were titrated and added at the start of MLR assays, consisting of a total of 5×10^4^ responder CD4^+^CD25^-^ T cells from the same donor of CD4^+^ regulator cells and 5×10^4^ γ-irradiated target PBMC from the donor of CD40-activated B cells or unrelated donor. Proliferation of responder was analyzed by [^3^H]-thymidine incorporation assay. [^3^H]-thymidine incorporation was expressed as the mean ± SEM counts per 1 min of 4 to 6 measurements.

### Blocking assays

Blocking studies were performed with a fusion protein ICOS-Ig (a gift from Anthony Coyle, Millennium Pharmaceutics), and the neutralization mAbs directly against CTLA-4 (10µg/ml, Ancell, USA) in conjunction with their relevant isotype controls. In some experiments, protease inhibitor 30µM of E64 and 100μM of pepstatin A (Calbiochem, Germany) were separately added at the initiation of co-culture and supplemented with the replacement of medium every three days.

### Statistical analysis

Data are means ± SEM. The differences in different treatment groups were analyzed by unpaired two-tailed Student’s *t* test following One-Way ANOVA test. Graphs and statistical analyses were performed with the use of Prism 5.0 for Windows software (GraphPad Software, San Diego, CA, USA). P values of 0.05 or less were considered significant.

## Results

### Expression of ICOS in CD4^hi^ Treg induced by allogeneic B cells

**Figure 1:**
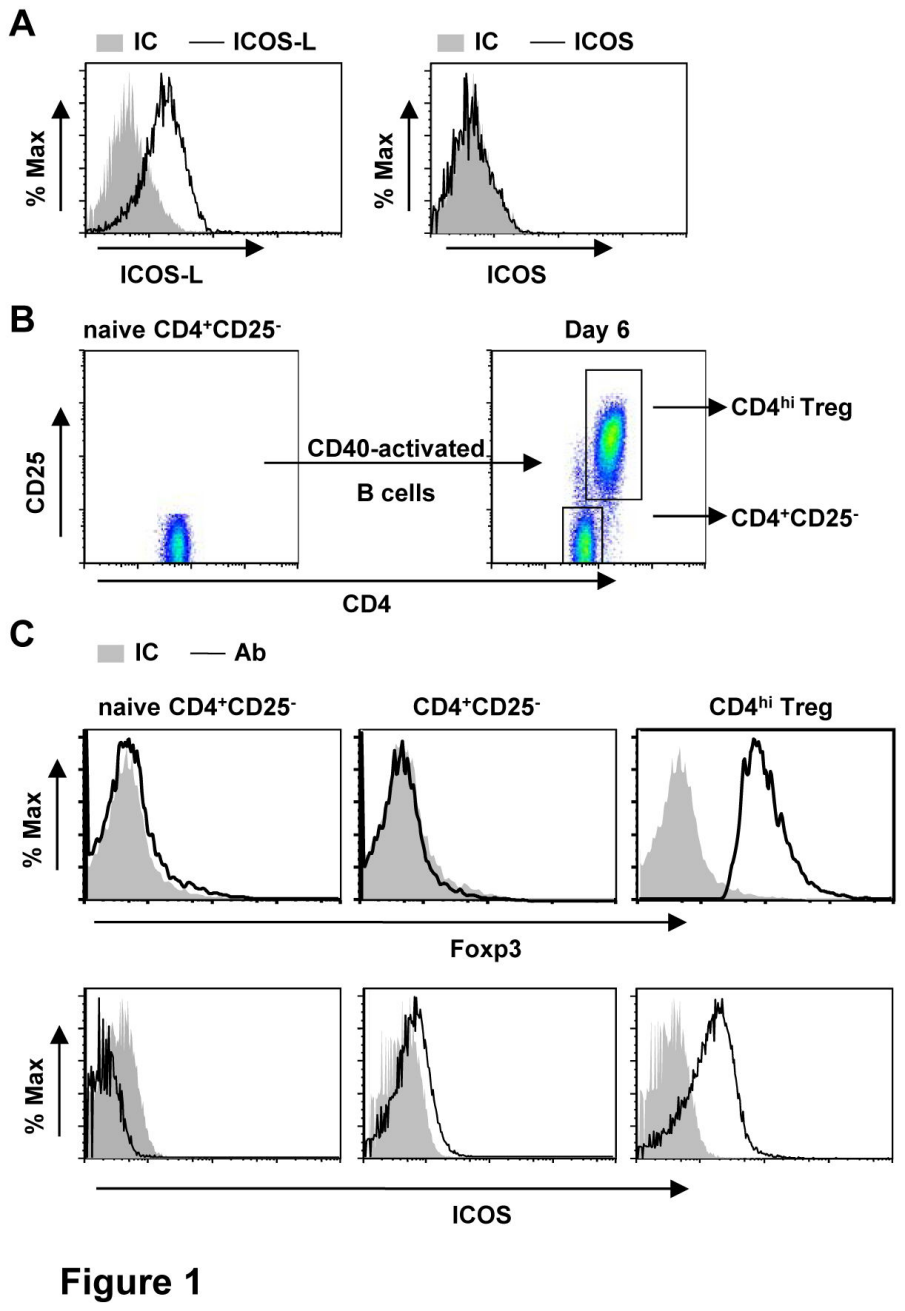
ICOS expressions in CD40-activated B cells and CD4^+^ T cell subsets. (A) Expressions of ICOS and ICOSL in CD40-activated B cells. (B-C) Naïve CD4^+^CD25^-^ T cells isolated from normal PBMC were co-cultured with allogeneic CD40-activated B cells at a ratio of 10:1 without exogenous cytokines for 6 days, the expressions of CD4, CD25 (B) and Foxp3, ICOS (C) in CD4^+^ T cell populations were determined by flow cytometry. The data represent for 20 independent experiments.

We firstly examined the expression of ICOS and ICOS-L in allogeneic CD40-activated B cells and T cells. As shown in Figure 1A, CD40-activated B cells expressed ICOS-L but did not express ICOS. Consistent with our previous results [33,34], a novel CD4^hi^CD25^+^ T cell subpopulation was induced from naive CD4^+^CD45RO^-^CD25^-^ T cells after 6 days of co-culture with allogeneic CD40-activated B cells (Figure 1B). This CD4^hi^CD25^+^ T cell subpopulation expressed high level of Foxp3 (Figure 1C) and thus was refered as CD4^hi^ Treg. Although ICOS expression was absent in naive CD4^+^CD25^-^ T cells, the induced CD4^hi^ Treg expressed high levels of ICOS after 6 days of co-culture (Figure 1C). These results suggest ICOS-ICOSL might be involved in the interaction between T and B cells.

### Blockade of ICOS-ICOSL impairs the generation and survival of CD4^hi^ Treg

**Figure 2:**
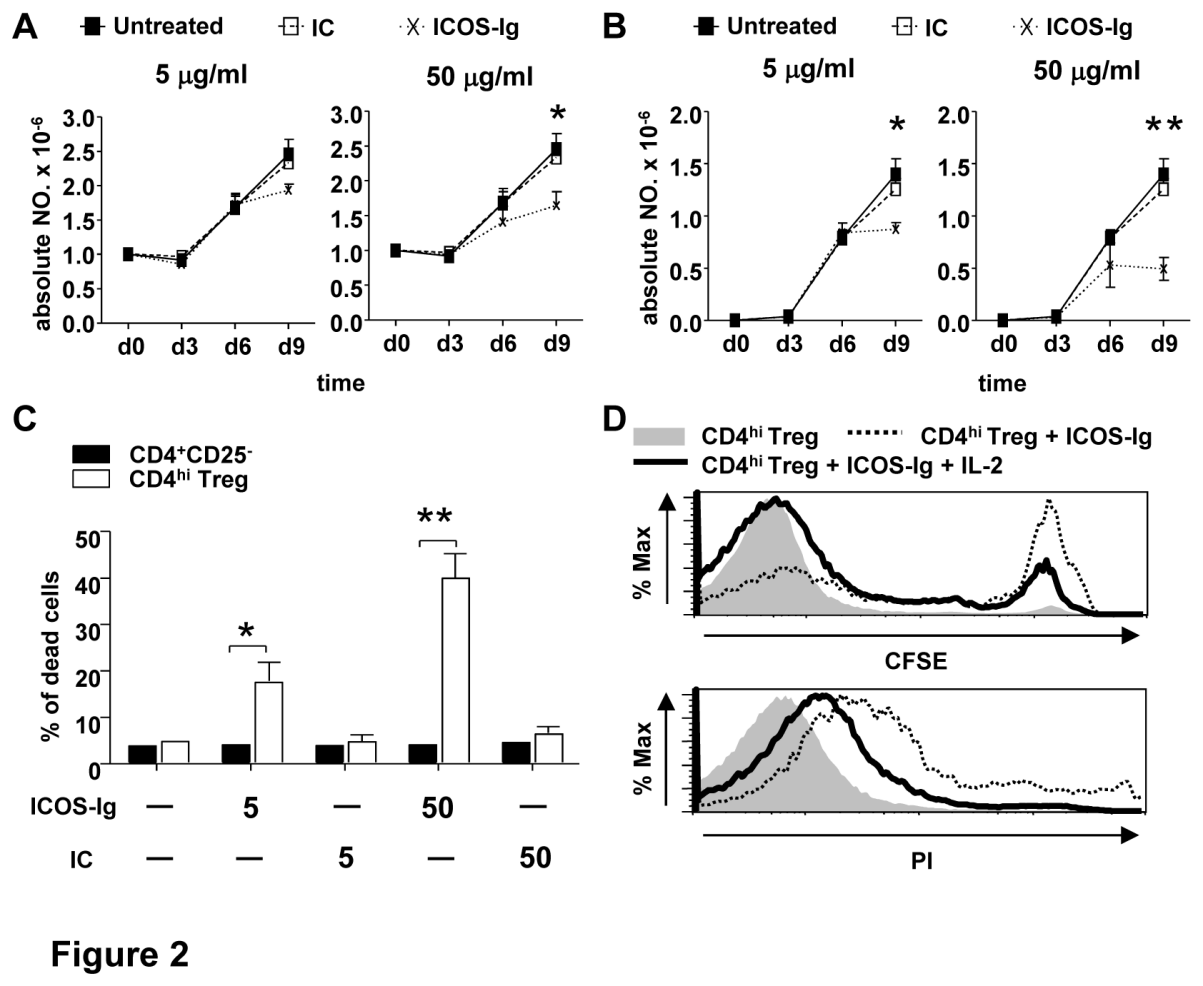
ICOS-ICOSL mediates the generation of CD4^hi^ Treg. Naïve CD4^+^CD25^-^ T cells were co-cultured with allogeneic CD40-activated B cells at a ratio of 10:1 for indicated time. ICOS-Ig and its relevant isotype controls (IC) were added at the final concentration of 5µg/ml or 50µg/ml since the initiation of co-culture and supplemented with replacement of medium every 3 days. The absolute number of total CD4^+^ T cells (A) and CD4^hi^ Treg (B) was examined. The percentage of PI^+^ cells in CD4^+^CD25^-^ and CD4^hi^ Treg on day 9 post co-culture (C) was examined as described in “cell death and proliferation assays”. Data for 4 different experiments are shown here and the 2-tailed unpaired Student t tests were used for comparing between group with ICOS-Ig and IC at indicated time. (n=4, *P<0.05, **P<0.01) (D) Exogenous IL-2 on proliferation and apoptosis of CD4^hi^ Treg. 50µg/ml ICOS-Ig was added into the co-culture with or without 250IU/ml recombinant human IL-2 and suppleted with replacement of medium every 3 days. The proliferation and apoptosis of CD4^hi^ Treg were determined by CFSE and PI staining correspondingly on day 9 post co-culture. Data represent for 3 independent experiments.

We next investigated the role of ICOS-ICOSL in the generation of CD4^hi^ Treg induced by CD40-activated B cells. The blockade of the ICOS-ICOSL interaction by ICOS-Ig fusion protein inhibited the generation of CD4^hi^ Treg in a dose dependent manner, as shown by the decreases in the absolute numbers of total CD4^+^ and CD4^hi^ Treg after 6 days of co-culture (Figure 2A and 2B). To understand the mechanisms underlying the decrease in the generation of Treg by blocking ICOS-ICOS-L interaction, we further examined the effect of ICOS-Ig on survival of CD4^+^ T cells during co-culture. As shown in Figure 2C, the treatment of ICOS-Ig significantly increased the cell death of CD4^hi^ Treg but did not affect the survival of CD4^+^CD25^-^ T cells. These results indicated that the ICOS-ICOSL interaction improves the generation and survival of CD4^hi^ Treg. Since IL-2 is an important down-stream molecule of ICOS-related signal pathway [1] while endogenous IL-2 secreted by CD40-activated B cells play cardinal role during generation of CD4^hi^ Treg [33], we then added IL-2 into co-culture to investigate whether the decreased generation of CD4^hi^ Treg could be reversed by exgenous IL-2. As shown in Figure 2D, the addition of IL-2 promoted the expansion of CD4^hi^ Treg while rescued their death induced by ICOS-Ig, which confirmed the crucial role of IL-2 in signal transferred by ICOS-ICOSL. 

**Figure 3:**
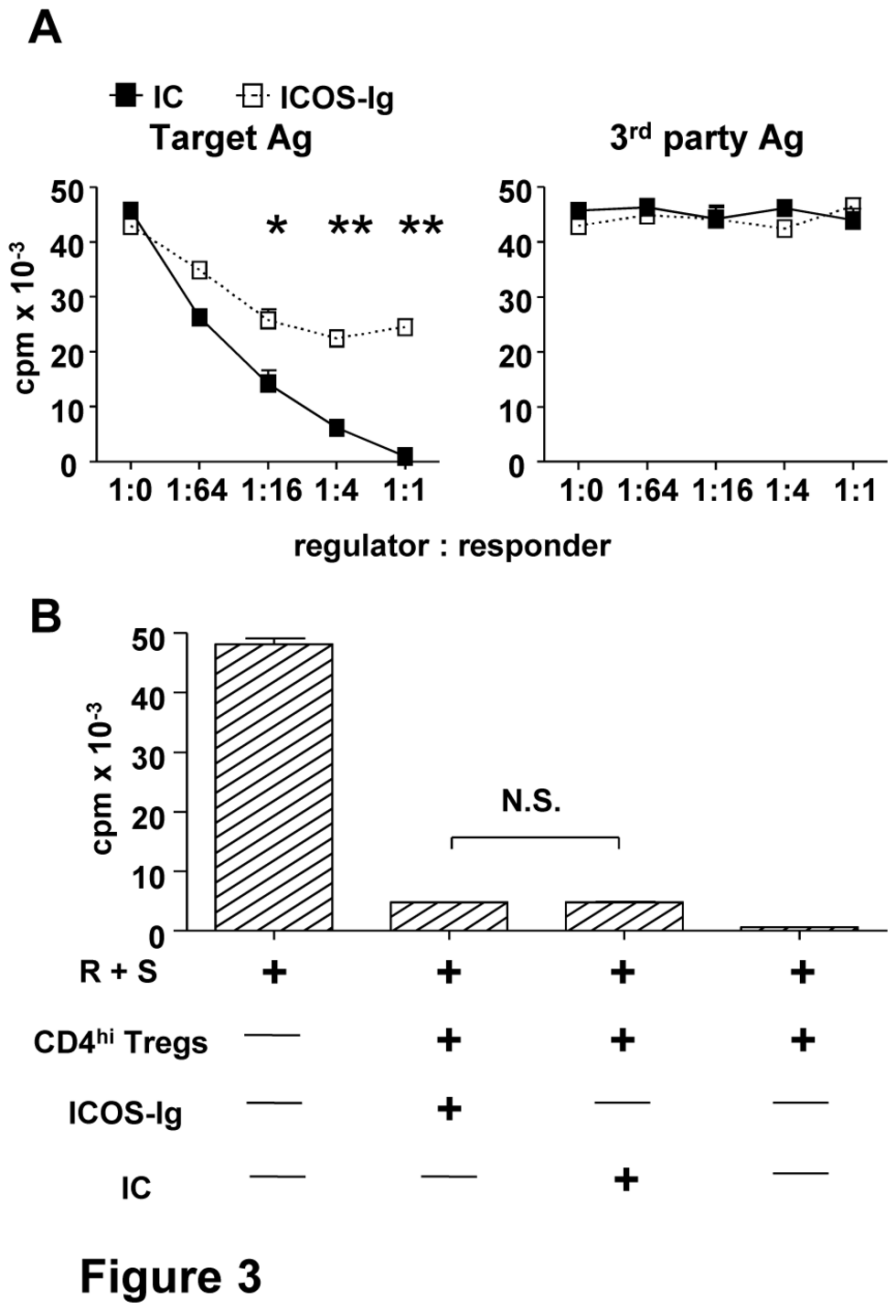
ICOS is not required for CD4^hi^ Treg effector function. CD4^+^CD25^–^ cells (5×10^4^, responder, R) and γ-irradiated allogeneic PBMC (5×10^4^, stimulator, S) were co-cultured as described in “MLR assays”. (A) CD4^hi^ Treg was sorted on day 9 from the co-culture supplemented with 50µg/ml ICOS-Ig or its relevant isotype controls (IC). The sorted cells were serially diluted and added into MLR as regulators. (B) Innocent CD4^hi^ Treg were used as regulators, and ICOS-Ig or IC was added respectively on the start of the MLR co-culture at the final concentration of 50µg/ml. On day 3 after co-culture, proliferation of responder was examined as described. The data represent for 4 independent experiments and the 2-tailed unpaired Student t tests were used for comparing between groups with ICOS-Ig and IC. (n=4, *P<0.05, **P<0.01).

ICOS is not required for CD4^hi^ Treg effector function To clarify whether the blockade of ICOS-ICOSL during the initial co-culture could affect the functions of induced CD4^hi^ Treg, we added ICOS-Ig and its control Ig (IC) at the start of the co-culture of naïve CD4^+^CD45RO^-^CD25^-^ T cells with allogeneic CD40-activated B cells. Consistent with our previous report [33], CD4^hi^ Treg induced by allogeneic CD40-activated B cells were alloantigen-specific Treg, since they only suppressed the original target alloantigen-induced proliferation but showed no effects on third-party alloantigen-induced proliferation (Figure 3A). The alloantigen-specific suppressive ability of CD4^hi^ Treg generated in the co-culture supplemented with ICOS-Ig decreased significantly compared to the control group (Figure 3A). We further added ICOS-Ig or control Ig (IC) directly into MLR system to illustrate whether ICOS participate into the suppression mediated by innocent CD4^hi^ Treg. However, the blockade of ICOS-ICOSL did not reverse CD4^hi^ Treg-mediated suppression on MLR (Figure 3B). These data suggest that ICOS-ICOSL plays a critical role in the generation of functional CD4^hi^ Treg but is not involved in effect phase of its suppression.

### Blockade of ICOS-ICOSL decreases Foxp3 and surface CTLA-4 expressions in CD4^hi^ Treg

**Figure 4:**
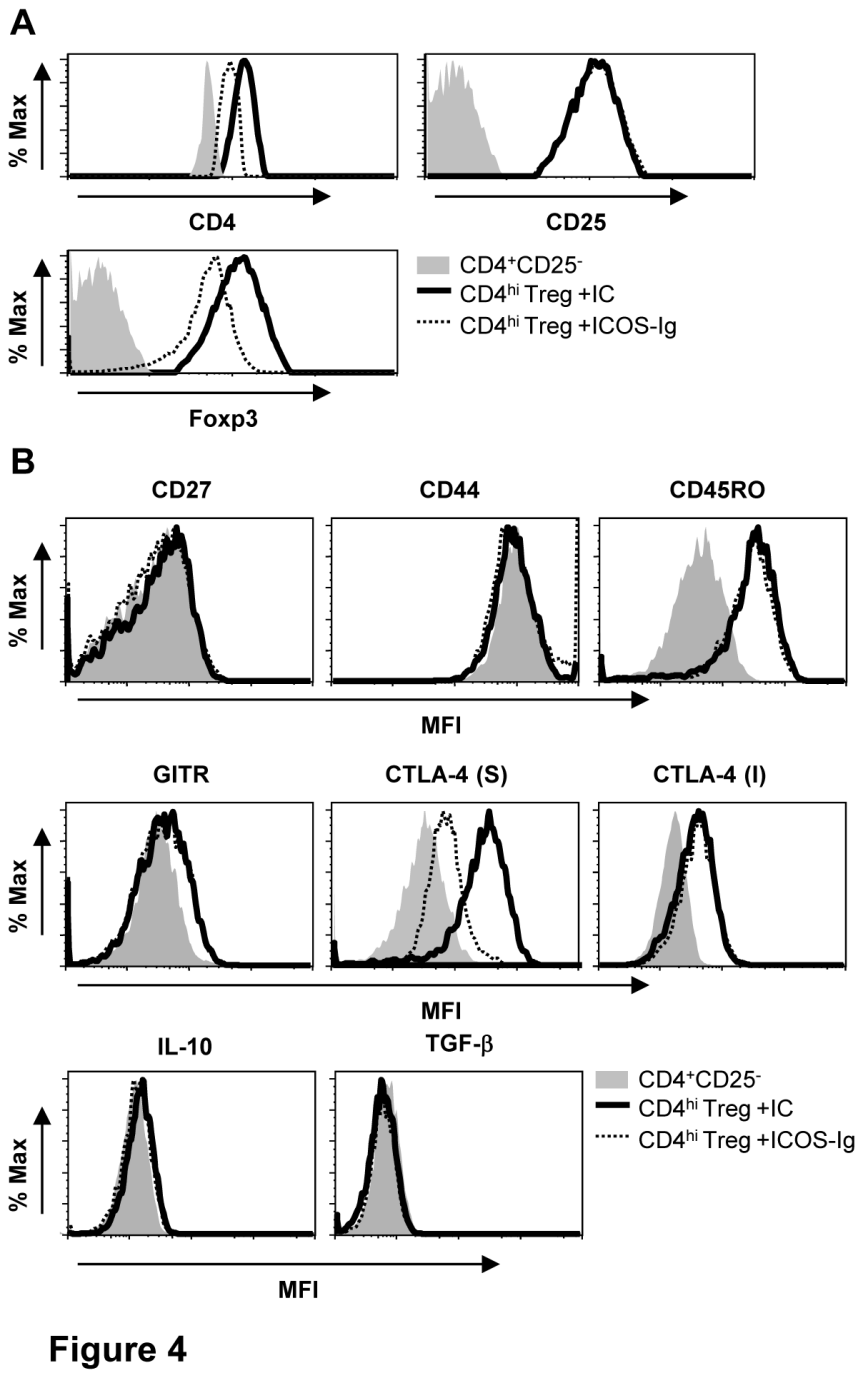
Blockade of ICOS-ICOSL affects the expressions of CD4^hi^ Treg markers. Naïve CD4^+^CD25^-^ T cells were co-cultured with allogeneic CD40-activated B cells at a ratio of 10:1 with the supplement of 50µg/ml ICOS-Ig or its relevant isotype controls (IC) for 9 days. (A) Expressions of CD4, CD25 (up panel) and Foxp3 (bottom panel) in CD4^+^CD25^-^ T cells and CD4^hi^ Treg on day 9 post co-culture. (B) Expressions of T cell activation markers CD27, CD44, CD45RO (up panel), Treg-related markers GITR, surface CTLA-4, whole-cell CTLA-4 (middle panel), intracellular inhibitory cytokines IL-10 and TGF-β (bottom panel) in CD4^+^CD25^-^ T cells and CD4^hi^ Treg on day 9 post co-culture. Data represent for 4 different experiments are shown.

To explore the mechanisms underlying the effects of ICOS on the generation of functional CD4^hi^ Treg, we firstly compared the expression levels of CD4, CD25, and Foxp3 in CD4^hi^ Treg induced by CD40-activated B cells with the addition of ICOS-Ig to those of control group. As shown in Figure 4A, blockade of ICOS-ICOSL decreased the expressions of CD4 and Foxp3, but did not affect CD25 expression in induced CD4^hi^ Treg. We then examined the expressions of T cell activation markers (CD27, CD44, and CD45RO), and found there were no differences of the expression for these activation makers in CD4^hi^ Treg between groups treated with ICOS-Ig or its control Ig (IC) (Figure 4B), suggesting these activation molecules are not involved in the ICOS-mediated CD4^hi^ Treg generation. The involvements of GITR, IL-10, and TGF-β in the impaired suppressive capacity of CD4^hi^ Treg caused by ICOS-Ig were also excluded because no difference of these molecules was found in CD4^hi^ Treg between groups treated with ICOS-Ig versus its isotype control Ig (Figure 4B). Interestingly, surface CTLA-4 expression in CD4^hi^ Treg generated in presence of ICOS-Ig decreased significantly compared to the control, whereas there was no difference in whole-cell CTLA-4 expression determined by antibody staining after fixation and permeabilization (Figure 4B). These data indicated that blockade of ICOS-ICOS interaction decreased Foxp3 and surface CTLA-4 expressions in CD4^hi^ Treg, suggesting the reduced Foxp3 and surface CTLA-4 expressions may contribute to the impaired suppressive capacity of CD4^hi^ Treg caused by blockade of ICOS-ICOSL. 

### Generation of functional CD4^hi^ Treg is dependent on its surface expression of CTLA-4

**Figure 5:**
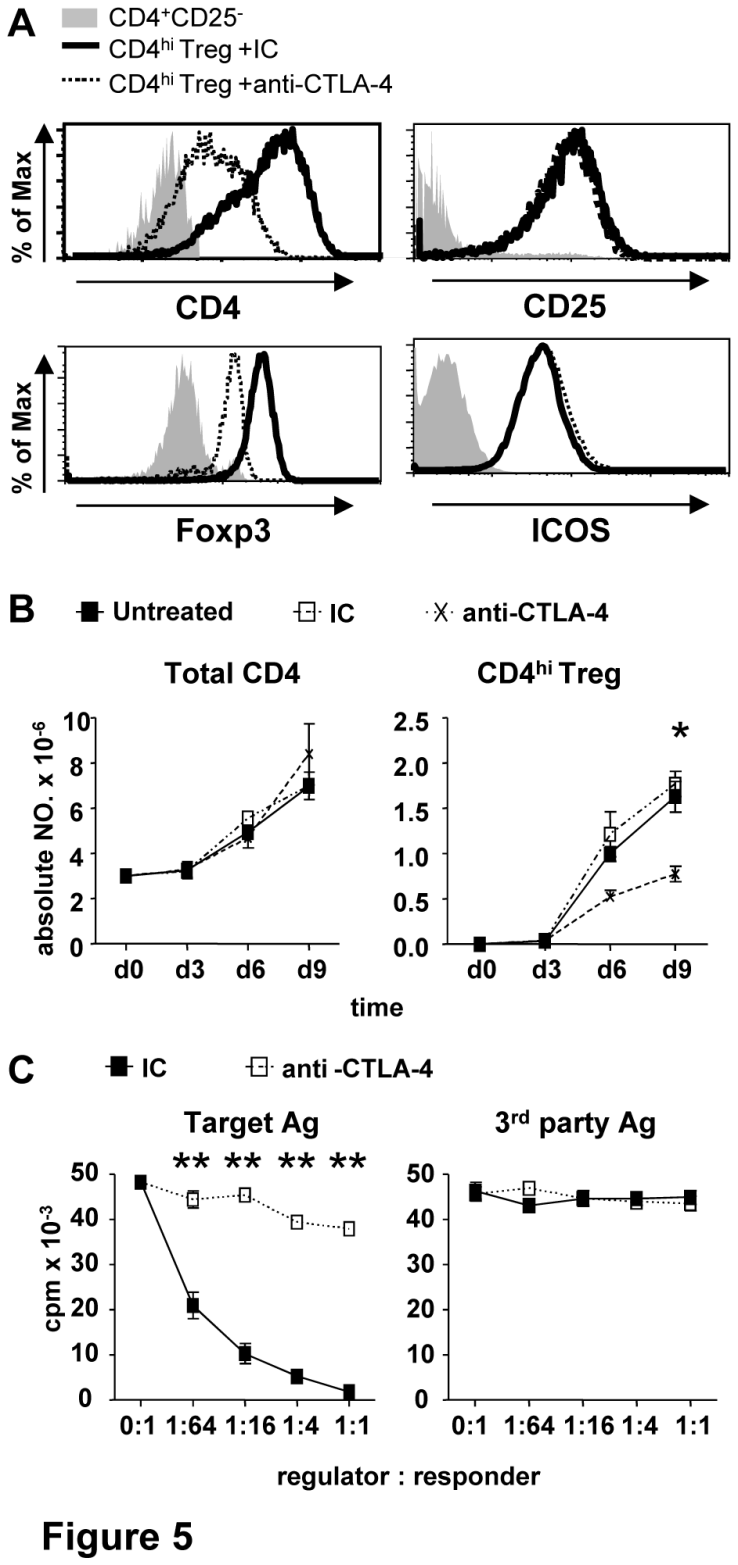
Surface CTLA-4 mediates the generation of functional CD4^hi^ Treg. Naïve CD4^+^CD25^-^ T cells were co-cultured with allogeneic CD40-activated B cells at a ratio of 10:1 in the presence of 10µg/ml CTLA-4 neutralizing mAb or its isotype control (IC). (A) Expressions of CD4, CD25 (up panel) and Foxp3, ICOS (bottom panel) in CD4^+^CD25^-^ and CD4^hi^ subsets from different treatment groups on day 6 post co-culture. (B) The absolute number of total CD4^+^ T cells (left panel) and CD4^hi^ Treg (right panel) from different treatment groups during 9 days. (C) Antigen-specific inhibition mediated by CD4^hi^ Treg from different treatment groups. CD4^+^CD25^–^ cells (5×10^4^, responder, R) and γ-irradiated allogeneic PBMC (5×10^4^, stimulator, S) were co-cultured while CD4^hi^ Treg from different groups were sorted on day 6 post co-culture and added into MLR system at different concentration. 3 days later, proliferation of responder was determined as described in “MLR assays”. Results shown here are representative of 4 independent experiments (n=4, *P<0.05, **P<0.01).

To confirm the benefit role of surface CTLA-4 expression in the generation of CD4^hi^ Treg, anti-CTLA-4 neutralizing mAb, which block the interaction between surface CTLA-4 and its ligand CD80/CD86, or its isotype control (IC) were added into the co-culture of naïve CD4^+^CD45RO^-^CD25^-^ T cells with allogeneic CD40-activated B cells at the start of incubation. Similar to ICOS-Ig, CTLA-4 neutralizing mAb down-regulated the expressions of CD4 and Foxp3 but did not affect the expression of CD25 or ICOS in the induced CD4^hi^ Treg (Figure 5A). Meanwhile, CTLA-4 neutralizing mAb significantly decreased the absolute number of the induced CD4^hi^ Treg (Figure 5B). Most importantly, the treatment of CTLA-4 neutralizing mAb abolished most of the alloantigen-specific suppression of induced CD4^hi^ Treg (Figure 5C). We also detected the effects of conjunction of anti-CTLA-4 and ICOS-Ig on the generation and function of CD4^hi^ Treg but found there was no significant difference in the outcome between ICOS-Ig+anti-CTLA-4 and anti-CTLA-4 alone group, (data not shown), suggesting that the function of ICOS was mainly dependent on the surface expression of CTLA-4. The indispensable role of CTLA-4 during the generation of functional CD4^hi^ Treg demonstrated here strongly suggests that surface CTLA-4 expression play a critical role in ICOS-mediated CD4^hi^ Treg generation.

### ICOS regulates the dynamic transfer of CTLA-4 between cell surface and cytoplasm

**Figure 6:**
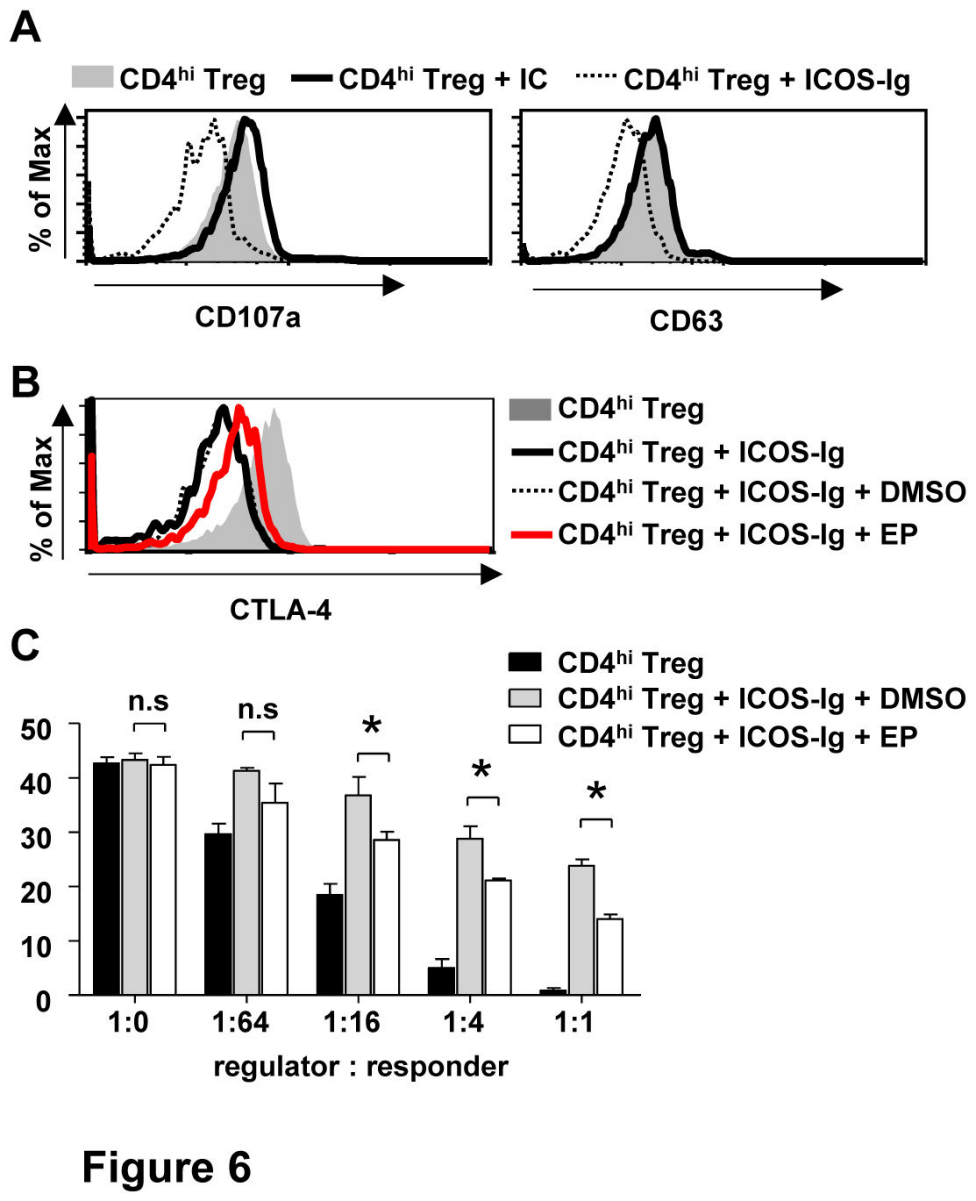
ICOS regulates the dynamic transfer of CTLA-4 between cell surface and cytoplasm. (A) The surface expressions of CD107a (left panel) and CD63 (right panel) on CD4^hi^ Treg were determined by FACS analysis after 9 days of co-culture with allogenic CD40-activated B cells at a ratio of 10:1 with 50ug/ml of ICOS-Ig or its relevant isotype controls (IC). (B-C) Inhibition of endocytosis restored the surface CTLA-4 expression and impaired suppressive capacity of CD4^hi^ Treg caused by ICOS-ICOSL blockade. The surface expression of CLTA-4 in CD4^hi^ Treg (B) were determined by FACS analysis after 9 days of co-culture with allogenic CD40-activated B cells at a ratio of 10:1 with 50µg/ml of ICOS-Ig or its relevant isotype controls (IC). 30µM of E64 and 100uM of pepstatin A (EP) solved in DMSO were added in the culture for inhibiting endocytosis. Antigen-specific inhibition mediated by CD4^hi^ Treg sorted on day 6 post co-culture from different treatment group (C) were determined as described in “MLR assays”. Results shown are representative of four independent experiments (n=4, *P<0.05, n.s. no significant difference).

Finally, we investigated the underlying mechanisms of the decreased expression of surface CTLA-4 in CD4^hi^ Treg induced with the addition of ICOS-Ig. Since total CTLA-4 expression was innocent with ICOS-Ig treatment, we supposed that the decreased surface CTLA-4 might resulted from its impaired transport between surface and cytoplasm. This hypothesis was supported by the decrease of exocytosis as evidenced by the reduced CD107a and CD63 expressions in CD4^hi^ Treg after ICOS-Ig treatment (Figure 6A). To restore the surface CTLA-4 expression in CD4^hi^ Treg, endocytosis inhibitors E64 and pepstatin A (EP) were added at the start of co-culture of naïve CD4^+^CD45RO^-^CD25^-^ T cells with allogeneic CD40-activated B cells. As shown in Figure 6B, decreased surface CTLA-4 expression caused by ICOS-Ig was reversed by the addition of protease inhibitor EP. Consistent with that, the suppressive capacity of CD4^hi^ Treg induced in the presence of ICOS-Ig was improved by EP treatment. These results indicate the decreased surface CTLA-4 expression and reduced suppressive capacity in CD4^hi^ Treg caused by ICOS-Ig were related to the impairment of CTLA-4 transport from cytoplasm to membrane. 

## Discussion

As a member of CD28 and CTLA-4 costimulator family, ICOS can be induced in activated T cells and mediates diverse immune reactions [1], but their roles in the generation of Treg, especially human Treg are still on debate [26,29,30]. We previously established a system to generate alloantigen-specific CD4^hi^ Treg from their naive precursors by co-culture with allogeneic CD40-activated B cells in vitro. However, the roles of co-stimulators in the generation of this induced Treg remain to be clarified. In this study, we demonstrated that ICOS-ICOSL was essential for the generation of CD4^hi^ Treg. More importantly, ICOS-related signal determined the generation of functional CD4^hi^ Treg through regulating the transport of CTLA-4 in CD4^hi^ Treg.

ICOS-ICOSL interaction is involved in human T cell activation and tolerance, and these functions were partly dependent on the induction of endogenous IL-2 [1,35]. Here we found that blockade of ICOS-ICOSL resulted in the decreased generation of CD4^hi^ Treg (Figure 2B), which was partly caused by the increased cell apoptosis (Figure 2C). Consistent with that, our previous study demonstrated that IL-2 secreted from CD40-activated B cells might play an important role during the generation of CD4^hi^ Treg [33]. Although it has been suggested that the response of CD4^+^ T cells to IL-2 could be limited because of low expression level of CD122 [3], high CD25 expression level of induced CD4^hi^ Treg (Figure 1) might compensate for this mechanism and make IL-2 indispensable in our system. The fact that ICOS^+^ CD4^+^ Treg was more sensitive to IL-2 than their ICOS^-^ analogues also strengthen the importance of IL-2 in the generation of CD4^hi^ Treg [24]. This hypothesis was supported by the facts that addition of exogenous human IL-2 suppressed apoptosis and restored the proliferation of CD4^hi^ Treg caused by ICOS-Ig (Figure 2D). 

It was found that suppressive capacity of induced CD4^hi^ Treg was also impaired by blockade of ICOS-ICOSL interaction during the generation of CD4^hi^ Treg (Figure 3A). Meanwhile, the reduced suppressive ability of CD4^hi^ Treg was found to be accompanied with the decreased expression levels of CD4, Foxp3 and surface CTLA-4 (Figure 4A and 4B). These results were consistent with the previous studies [33,36], in which Foxp3 and CTLA-4 were confirmed to be vital for the suppression mediated by CD4^+^ Treg. However, the effect of ICOS-ICOSL was only identified during the generation of CD4^hi^ Treg, but not directly involved in CD4^hi^ Treg-mediated suppression on allo-responses (Figure 3B).

CTLA-4 is a critical inhibitory molecule competing for CD80/CD86 with CD28 [37,38], and plays an essential role in Treg-mediated suppression both in vivo and in vitro [39-41]. Both CTLA-4 and ICOS are important co-stimulatory molecules [42], but the interaction between these two pathways is still unclear. Although blockade of CTLA-4 was found to promote the expansion of T cells mediated by stimulating intrinsic expression of ICOS [43,44], its effect on ICOS expression here was indispensible because of high ICOS expression in CD4^hi^ Treg (Figure 5A). Importantly, it has never been reported whether ICOS could regulate the expression of CTLA-4 in reverse. Here we for the first time demonstrated that blockade of ICOS-ICOSL interaction caused decreased surface CTLA-4 expression in CD4^hi^ Treg without influencing its whole-cell expression level (Figure 4B). Generally, CTLA-4 is expressed on cell surface transiently and then recycled readily through endocytosis initiated by the attachment of adapter protein 2 (AP-2) on its cytoplasmic tail [32]. However, the interaction of AP-2 with CTLA-4 could be replaced by activated phosphatidylinositol 3-kinase (PI3K), which could result in decreased endocytosis of CTLA-4 [45,46]. Since surface CTLA-4 and ICOS generally gathered around activated TCR, PI3K recruited to ICOS might compete with AP-2 on the link locus of CTLA-4 and thus protect CTLA-4 from endocytosis. This hypothesis was partly supported by the reverse of decreased surface CTLA-4 expression and reduced suppressive capacity in CD4^hi^ Treg caused by ICOS-Ig with the treatment of endocytosis-related protease inhibitors EP (Figure 6B). On the other side, cytoplasm CTLA-4 was reported to localize in lysosome or lysosome-related organelles (LRO), and exported back to surface by exocytosis [47,48]. Here we also found that surface expression levels of two lysosome markers CD107a and CD63 decreased accompanied with CTLA-4 with the treatment of ICOS-Ig (Figure 6A), which suggested that blockade of ICOS-ICOSL reduced exocytosis as well. Our results exhibited a novel relationship between CTLA-4 and ICOS through molecule transport between cell membrane and cytoplasm, which might be generally involved in self-regulation of CD4^+^ T cells generally and deserve further investigation.

In addition to CTLA-4, Foxp3 is also regarded as a characteristic marker of Treg, and the expression level of Foxp3 might represent for the suppressive capacity of Treg [36]. Although there have been several reports suggesting that the expression of Foxp3 can control the expression of CTLA-4 in Treg [36,49,50], the influences of CTLA-4 on the expression of Foxp3 has never been reported. Here we found that, similar to treatment with ICOS-Ig, blockade of CTLA-4 decreased the expressions of CD4 and Foxp3 but not CD25 (Figure 5A). More importantly, the decreased expression of Foxp3 was accompanied with the impaired suppressive capacity of CD4^hi^ Treg (Figure 5C). Another interesting point was the neutralization of CTLA-4 signal impaired only the expansion of induced CD4^hi^ Treg but did not affect the absolute number of total CD4^+^ T cells (Figure 5B), which suggested that CTLA-4 determine the quality rather than quantity of CD4^hi^ Treg. 

In summary, our study demonstrated some important effects of ICOS-related signals in the generation of a novel human alloantigen-specific CD4^hi^ Treg. Moreover, this investigation provides some interesting information on the interplay of co-stimulators and their effects on the expression of Foxp3 and inhibitory functions of CD4^+^ Treg, which undoubtedly improve our understanding in underlying mechanisms of T cell differentiation and self-regulation, and offer some important molecular basis for future Treg-based therapy.
